# Is social exposure to obesity associated with weight status misperception? Assessing Australians ability to identify overweight and obesity

**DOI:** 10.1186/s12889-019-7556-9

**Published:** 2019-09-04

**Authors:** C. A. Opie, K. Glenister, J. Wright

**Affiliations:** 1 Department of Rural Health, The University of Melbourne, Graham Street, Shepparton, Victoria 3630 Australia; 2 Echuca Regional Health, Research Department, 226 Service Street, Echuca, Victoria 3564 Australia

**Keywords:** Obesity, Overweight, Body mass index, Waist circumference, Social conditioning, Weight perceptions

## Abstract

**Introduction:**

Overweight and obesity prevalence has increased significantly over the past two decades, currently impacting greater than 60% of Australians. It is unclear if a social perception of a healthy weight has been obscured by the increase in prevalence and thus has become inconsistent with the medical definitions.

**Methods:**

An electronic questionnaire was distributed via email and social media using the authors’ informal networks. Australian adults were eligible to participate. Participants were asked to categorise their own body size using medically accepted words and previously published silhouettes, before identifying underweight, healthy weight, overweight or obesity in a series ofsilhouettes.

**Results:**

Eight hundred six questionnaires were completed, a majority of participants had attained a high level of education and were employed female health professionals. Under half the studied population had a Body Mass Index (BMI) corresponding to overweight or obese categories (*n* = 349, 47%). Accuracy in self-perceived weight status using medicalised words was higher among respondents with BMI corresponding to the healthy weight category (*n* = 311, 85%) and overweight category (*n* = 133, 74%) than for respondents with BMI corresponding to obesity (*n* = 79, 45%) or underweight (*n* = 5, 31%). A majority of respondents were able to accurately self-perceive their weight status using silhouettes (*n* = 469, 70%). Females were significantly more likely to be accurate in their self-perception than males, using both medicalised words (*p* = < 0.001) and silhouettes (*p* = 0.045). Respondents with a BMI corresponding to the obese category were significantly more likely to be accurate with weight status self-perception using silhouettes than words (87% versus 46% respectively, *p* = < 0.001). Less than half (41%) of respondents accurately perceived silhouettes corresponding to an overweight BMI and less than one in ten respondents (9%) accurately perceived the lower limit of the silhouettes corresponding to an obese BMI.

**Conclusions:**

Repondents were challenged to accurately perceive silhouettes corresponding to an obese BMI in themselves and others. Weight status misperception was more likely to exist among those with a BMI less than 18.5 or 30 or more (underweight BMI and obese BMI). Accuracy decreased as BMI increased. Respondents with a BMI in the obese category were significantly more likely to accurately self-perceive their weight status using silhouettes than medicalised words. Silhouettes may act as an effective visual cue in initiating weight related discussions.

**Electronic supplementary material:**

The online version of this article (10.1186/s12889-019-7556-9) contains supplementary material, which is available to authorized users.

## Introduction

Australia is experiencing an obesity epidemic, directly and indirectly costing the economy a conservative estimate of $8.6 billion (2014/15), a figure forecast to reach $87.7 billion by 2025 if not addressed [1]. In 2014–15, almost two out of three Australians (11.2 million people, 63%) were overweight or obese, a significant increase since 1995 (56%) [2]. Unhealthy weight increases the risk of chronic conditions including some forms of cancer, eating disorders, poor mental health, osteoarthritis, Type 2 Diabetes Mellitus (T2DM) and Cardiovascular Disease (CVD) [3, 4]. Overweight and obesity prevalence is even greater in rural populations (69%) [5].

Early intervention and secondary prevention of an unhealthy weight relies on Australian primary healthcare professionals routinely calculating body mass predictors (height and weight to calculate a Body Mass Index (BMI) and waist circumference), on every adult [4, 6]. Criticisms have been raised regarding the use of BMI due to the inability to assess proportions of muscle mass compared with fat mass [7], though when combined with waist circumference, it is considered a strong predictor of health risk [8]. Eighty seven percent of Australians access their General Practitioner (GP) at least once a year [9], emphasising that GPs are well poised to identify an unhealthy weight and treat accordingly [4, 8]. Furthermore, patients expect their GP to initiate a weight related discussion when required [10]. Yet, contemporary evidence suggests that less than a quarter of Australian adults (22%) have a BMI documented in their GP medical record and even fewer have a waist measurement recorded [11]. Morevoer, obesity is the individual problem managed in a patient encounter in 0.8% of GP consultations (0.8%) [6].

An unhealthy weight is however, a risk factor for many of the most common presenting conditions in general practice including musculoskeletal encounters (12% of GP activity), circulatory encounters (10%) or endocrine and metabolic encounters (9%) and therefore may provide a supplementary opportunity for brief intervention [6]. Although average appointment times are brief (approximately 15 min) [6], the majority of GP encounters involve a single issue and it is important to highlight the contribution of weight to relevant presenting complaints. Any opportunity for brief intervention (30 s) to motivate weight loss is known to be helpful and acceptable to over 99% of patients with obesity, even when the encounter is unrelated to weight [12]. Incidently, Ling and colleagues report that for adolescents, health professional recommendations are more likely to influence weight loss attempts than parents [13].

In a society where more people exhibit an unhealthy weight than a healthy weight, social perception of a healthy weight is at risk of becoming obscured [14], leading to people with overweight and/or obesity perceiving their weight as healthy. If people with overweight and/or obesity do not recognise their weight as a risk factor for chronic conditions, public health messages will not resonate. Binkin et al. (2013) reported in their obesity perception study among mothers and children that *‘what is common has a greater likelihood of being perceived as normal’* [15]. Misperception develops as early as 9 years of age and is heavily influenced by peer and familial weight [16]. Among adults, males are reportedly more likely to misperceive an overweight or obese weight status than females, regardless of cultural background [17]. Any misperception among society may be cause for concern, considering that it is believed that an individual attempt to lose weight is mainly driven by a self-perception of being “too heavy”, regardless of accuracy [13]. Health professionals are not immune to weight status misperception [10, 14, 18, 19] hence, poor identification of an unhealthy weight by health professionals may mislead or contribute to misperception.

There is limited evidence investigating whether adult Australians may be becoming obscured in their perceptions of weight status by the increase in prevalence of overweight and obesity. The aim of this study was to assess whether Australians, including health professionals are challenged to identify overweight or obesity and whether rural residence, body size or gender impacts identification.

## Method

### Study aim

To assess people’s ability to identify overweight and obesity.

### Study design

An electronic questionnaire was developed for this study, utilising silhouette images previously published [20] (Additional file 1). The questionnaire was pilot tested (self-administered, paper based) for clarity of questions and acceptability (*n* = 12) before being distributed as an electronic survey. Respondents were asked a series of demographic questions (age, gender, postcode, educational attainment, employment status and whether they were a health professional (HP). Respondents were asked to report their height and weight before matching their own weight with medicalised words (underweight; healthy weight; overweight; obese). Respondents were asked to match their own weight with gender specific body figure silhouettes [20] before matching each medicalised word with a silhouette. These silhouettes were developed by Harris et al. [20] as a novel pictorial method for assessing perception of weight status, and have been assessed for validity and test-retest reliability [20]. This study utilised these silhouettes to assess perception of weight status in both self and others, with the aim of identifying demographic variables associated with perception of weight status.

### Participants

Adults (aged 18 years and over) residing in Australia were eligible to participate.

### Recruitment

Primary dissemination of the online survey was through the researchers’ informal networks including their rural health service and university communities, via email and social media. Responders were asked to further disseminate the survey through their professional and personal networks as a snowballing recruitment strategy. Consent to participate was requested at the beginning of the online survey, unless the participant selected ‘yes’, they were unable to enter the survey. Data were collected and managed using RedCap electronic data capture tools [21] hosted at the University of Melbourne. RedCap (Research Electronic Data Capture, Vanderbuilt University, United States) is a secure, web-based application designed to support data capture for research studies, providing: 1) an intuitive interface for validated data entry; 2) audit trails for tracking data manipulation and export procedures; 3) automated export procedures for seamless data downloads to common statistical packages; and 4) procedures for importing data from external sources [21].

### Analysis

Self-reported height and weight data was used to calculate BMI according to the internationally defined formula: weight (kilograms) divided by the square of height (centimetres), before being categorised into underweight (BMI < 18.5); healthy weight (BMI 18.5–24.9); overweight (BMI 25–29.9); obese (BMI ≥ 30) [22]. Postcodes were categorised according to the Modified Monash Model into major cities (MMM1) and MMM2–7 (regional and remote) [23]. Data were analysed using Statistical Package for Social Sciences (SPSS) Version 22 for descriptive statistics and cross-tabulations between categories. Direct logistic regression was used to assess the impact of independent variables (age, sex, BMI, HP/non HP and city/rural residence) on accuracy of weight perception. Specifically, four separate logistic regression analyses were completed for the dependent variables of accurate perception of BMI category in self using words, accurate perception of BMI category in self using silhouettes, accurate perception of overweight in others (silhouettes) and accurate perception of obesity in others (silhouettes).

### Funding

Nil specific funding was sought for this study. All authors were employed by the The University of Melbourne, Department of Rural Health which is funded to conduct health research by the Commonwealth of Australia Rural Health Multidisciplinary Training Program.

## Results

A total of 806 questionnaires were completed, a majority of responders were female (*n* = 656, 85.1%), over half were HPs (*n* = 379, 50.8%) and of those that provided a postcode (*n* = 770, 95.5%), 64.4%, (*n* = 519) lived in a regional/remote community and 32.6% (*n* = 251) resided in a major city. On average participants were 41.0 ± 12.5 years old, a high rate of tertiary education and employment status is evident (Table 1).

**Table 1:** Education and Employment characteristics of the sample

Variable	n (%)
Education Status	
Partial Secondary	15 (2.0%)
Secondary	53 (6.9%)
Technical and Further Education (TAFE)	108 (14.1%)
Tertiary	590 (77.0%)
Employment Status	
Full Time	401 (49.8%)
Part Time	290 (36.0%)
Unemployed	8 (1.0%)
Student	85 (10.5%)
Retired	5 (0.6%)
Not in Labour Force	6 (0.7%)

### Body mass index

Height and weight was self-reported by 747 (92.7%) respondents; hence BMI was able to be calculated in the majority of cases (Table 2). Overweight prevalence was 23.7% (*n* = 177) and obesity prevalence was 23.0% (*n* = 172) in this cohort. The prevalence of obesity was similar among regional/remote respondents (*n* = 117, 24.1%) and respondents from major cities 23.0% (*n* = 56, 23.0%, *p* = 0.736).

**Table 2:** Self-reported Body Mass Index

	Male n (%)	Female n (%)	Sample n (%)	p (proportion males vs females χ^2^)
Underweight (BMI < 18.5)	1 (0.9%)	15 (2.4%)	16 (2.2%)	0.528
Healthy weight (BMI 18.5–24.9)	53 (48.6%)	312 (50.2%)	365 (50.0%)	0.755
Overweight (BMI 25–29.9)	32 (29.4%)	145 (23.3%)	177 (24.2%)	0.177
Obese (BMI ≥30)	23 (21.1%)	149 (24.0%)	172 (23.6%)	0.325

### Self-perception of weight status using medicalised words

Overall, 72% (*n* = 528) of respondents correctly identified the category that matched their BMI as calculated from self-reported height and weight. Accuracy was greatest for respondents with a BMI corresponding with the healthy weight category (*n* = 311, 85%) and overweight category (*n* = 133, 74%) and lower for respondents with a BMI corresponding with the underweight category (*n* = 5, 31%) and obese category (*n* = 79, 45%) (Table 3). Females were more commonly able to correctly categorise their own body size than males (73% vs 64% respectively, *p* < 0.001).

**Table 3:** Summary of self-perception of weight status using medicalised words versus silhouettes

	Medicalised Words				Silhouettes				Words vs Silhouettes
	Matched n (%)	Underestimated n (%)	Overestimated n (%)	*p* value	Matched n (%)	Underestimated n (%)	Overestimated n (%)	*p* value	*p* value
Overall	528 (71.9)	148 (20.2)	58 (7.9)	–	469 (70.2)	84 (12.5)	116 (17.3)	–	0.450
Male	70 (64.2)	35 (32.1)	4 (3.7)	< 0.001	60 (60.6)	19 (19.2)	20 (20.2)	0.045	0.591
Female	455 (73.4)	111 (17.9)	54 (8.7)		407 (72.0)	65 (11.5)	93 (16.3)		0.602
Regional/Remote	342 (70.4)	102 (21.0)	42 (8.7)	0.369	313. (69.6)	51 (11.3)	86 (19.2)	0.110	0.712
Major City	184 (75.4)	44 (18.0)	16 (6.6)		155 (72.4)	31 (14.5)	28 (13.0)		0.468
HP	264 (73.7)	66 (18.4)	28 (7.8)	0.552	235 (71.6)	39 (11.9)	54 (16.3)	0.763	0.538
Non-HP	248 (70.9)	76 (21.7)	26 (7.4)		223 (69.5)	44 (13.7)	54 (16.8)		0.695
Underweight	5 (31.3)	0	11 (68.8)	< 0.001^a^	8 (50)	0	8 (50.0)	< 0.001^a^	0.280
Healthy weight	311 (85.2)	10 (2.7)	44 (12.1)		258 (79.1)	23 (7.1)	45 (13.8)		0.037
Overweight	133 (74.3)	43 (24.0)	3 (1.7)		60 (37.3)	39 (24.2)	62 (38.5)		< 0.001
Obese	79 (45.5)	95 (54.6)	0		143 (86.7)	22 (13.3)	0		< 0.001

^a^Matched vs not matched

### Self-perception of weight status using silhouettes

Overall, 70.2% (*n* = 469) of respondents accurately perceived the silhouette that matched their BMI. Females (*n* = 407, 72%) were more likely to accurately perceive their silhouette than males (72% vs 61% respectively, *p* = 0.045). A significantly greater proportion of respondents with a BMI corresponding with the obese category self-perceived their weight status accurately using the silhouettes than the medically accepted words (87% vs 46%, *p* < 0.001) (Table 3).

### Weight status perception in others

**Fig. 1:**
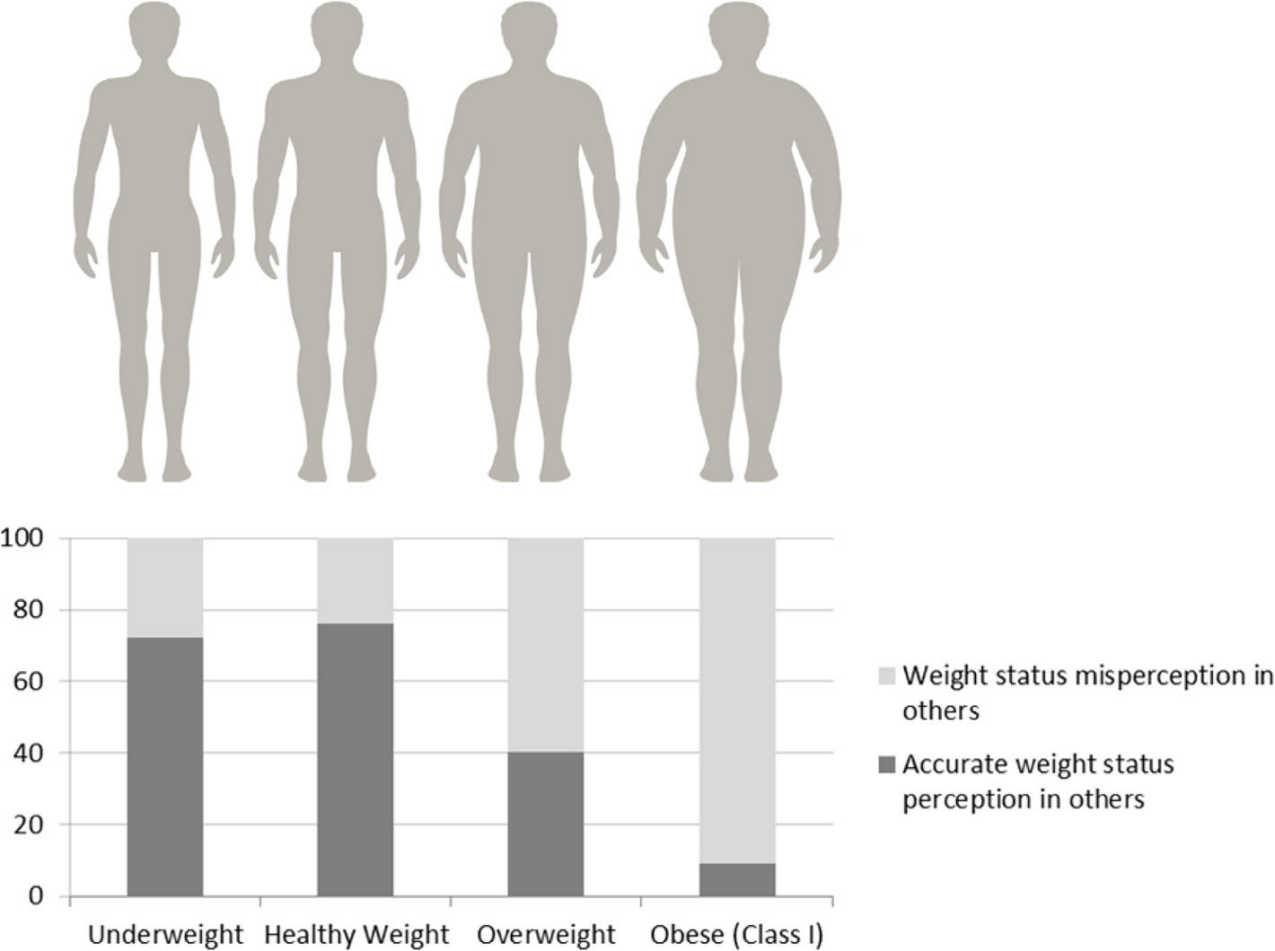
Perception of weight status in others using silhouettes (image sourced from iStock)

The majority of respondents accurately perceived the underweight silhouettes (*n* = 557, 69.1%) and healthy weight silhouettes (*n* = 589, 73.1%). Less than half of respondents accurately identified the overweight silhouette (283, 41%) and less than one out of ten respondents accurately perceived the lower limit of obesity within the silhouettes (65, 9.3%) (Fig. 1).

The ability to accurately perceive the lower limit of obesity did not differ significantly between males and females (7.1% vs 8.7%, *p* = 0.587), HPs and non-HPs (9.2% vs 7.6%, *p* = 0.430), respondents from regional/remote areas and cities (9.4% vs 6.4%, *p* = 0.151) or respondents with a BMI corresponding to obesity or not (8.6% vs 8.6%, *p* = 0.979). Four separate logistic regression analyses were undertaken (Table 4):

- Dependent variable: accurate perception of BMI category in self using words. As BMI increased accuracy decreased. Males were less accurate in perception of their own BMI category (odds ratio 0.41) than females.
- Dependent variable: accurate perception of BMI category in self using silhouettes. As BMI increased, accurate perception of own BMI category using silhouettes also increased. As age increased, accurate perception of own BMI category decreased.
- Dependent variable: accurate perception of overweight in others using silhouettes. Males were less accurate (odds ratio 0.46) than females in perception of overweight in others. As BMI increased perception of overweight in others decreased.
- Dependent variable: accurate perception of obesity in others using silhouettes. None of the independent variables included were significantly associated with accurate perception of silhouettes corresponding to obesity.

**Table 4:** Accurate perception of weight status, logistic regression (odds ratio, 95% CI, p value)

	Words	Silhouettes		
Independent variables:	Perception of weight status (self)	Perception of weight status (self)	Perception of overweight in others	Perception of obesity in others
Sex (male = 1, female = 0)	0.41	0.74	0.46	0.84
	0.25–0.66 *p* < 0.001	0.41–1.35 *p* = 0.327	0.29–0.74 *p* = 0.001	0.38–1.82 *p* = 0.653
BMI (continuous)	0.91	1.06	0.96	1.01
	0.89–0.94 *p* < 0.001	1.02–1.10 *p* = 0.006	0.94–0.99 *p* = 0.002	0.97–1.05 *p* = 0.424
Age (continuous)	1.00	0.96	0.99	0.99
	0.98–1.01 *p* = 0.758	0.95–0.98 *p* < 0.001	0.98–1.01 *p* = 0.304	0.97–1.01 *p* = 0.424
Health professional (yes = 1, no = 0)	1.03	1.18	0.81	1.15
	0.68–1.56 *p* = 0.879	0.74–1.67 *p* = 0.482	0.59–1.11 *p* = 0.190	0.67–1.99 *p* = 0.613
City (yes = 1, no = 0)	1.23	1.24	0.89	0.61
	0.78–1.95 *p* = 0.373	0.74–1.87 *p* = 0.428	0.63–1.26 *p* = 0.510	0.32–1.15 *p* = 0.128
Cases correctly classified by model	76.8%	81.9%	61.9%	91.3%

Females were significantly more accurate in self-perception of weight status than males using words. BMI appeared to influence accuracy of weight status in self and identification of a silhouette corresponding with an overweight BMI, though the association was weak. Females were significantly more accurate for perception of an overweight BMI category in others, than males. None of these variables were significantly associated with accuracy of identifying a silhouette corresponding with an obese BMI.

## Discussion

Overweight and obesity prevalence among self-reporting study respondents was lower than current Australian estimates (46.7% versus 63.4% respectively) [2]. Health literacy levels may be assumed to be high, considering the sample were well educated and mostly employed health professionals. Yet, a majority of respondents were unable to identify the lower limit of a BMI corresponding with the obese category, in both themselves and others. In fact, as weight status increased, accuracy decreased. The national obesity epidemic, where almost two out of three people are either overweight or obese [2] may have engendered a society that no longer recognises the threshold of unhealthy weight.

Men in particular lacked accuracy in self-perception of weight status using medicalised words (underweight; healthy weight; overweight; obese) and were more likely to underestimate their weight status using silhouettes than females. Men’s inability to accurately perceive their weight status using either medicalised words or visual cues has some support in the literature. A study from the United States (US), found that men with an overweight weight BMI perceived that they had a BMI corresponding with an underweight or a healthy weight status [24].

Weight status misperception using medicalised terminology was greatest among respondents corresponding with each end of the weight staus index (underweight and obese), regardless of gender. People at greatest risk of chronic ill health due to their weight therefore appear to have the poorest self-perception of their weight. In their cross-sectional survey of adolescents in Hong Kong, Cheung et al. (2007) found that females in particular are motivated to adopt weight control behaviours if they perceive themselves to be overweight [25]. Assuming active weight control behaviours resulting from weight self-perception in adolescents continue into adulthood, consistent attempts to measure weight predictors and discuss the effects of weight upon health is likely to be beneficial throughout life.

Respondents with a BMI corresponding with the obese category were however, more accurate in self-perception of weight status using silhouettes than medicalised words, a finding consistent with Harris et al. 2008. Self-perceiving an unhealthy weight is known to be most problematic among people with a BMI equating to overweight or obesity [20, 26]. Surprisingly however, regardless of weight status, almost nine out of ten respondents misperceived the lower limit of obesity using silhouettes, a finding that suggests that increasing prevalence of obesity and social exposure may indeed be influencing social perception of obesity [18].

Healthy weight people are now a minority in Australian society potentially influencing the perception of both HPs and the general population alike. HPs and non-HP respondents equally misperceived the lower limit of obesity using either medicalised terminology or silhouettes. Wong and colleagues (2016) found that medically trained professionals and the general public were mutually inaccurate in their perceptions of people identified as overweight or obese [19]. A recent US study found that parents underestimated the weight of their children with an overweight or obese BMI (96 and 72% respectively) and this pattern appeared independent of socioeconomic/educational factors [27]. The authors attributed the findings to social and cultural beliefs and attitudes that have resulted from the high incidence of children identified as overweight or obese in contemporary society in comparison to previous generations [27]. As it appears that weight related perceptions develop early in life, weight concerns ought to be addressed as early as possible, particularly in rural areas where prevalence is greater [5].

It is important to note the identified limitations of this study. Firstly, the responding adults in the study may be subject to selection bias and findings cannot be generalised to the Australian population, though may be representative of Australians with a mid to high socioeconomic status. The online survey was primarily disseminated within regional health services where the authors are situated, presumably contributing to the high representation of English speaking females with internet access, health professionals with a higher education and regional/remote people. If the sample was more representative of the Australian population and included a higher proportion of males, people with low socioeconomic status and low educational attainment, then perception of overweight and obesity may be able to be more fully explored among people at particular risk of obesity. Secondly, results are subject to social desirability bias where it is known that people may report an inaccurate height and weight in an attempt to impress and conform with social norms [28], though underestimation of weight and overestimation of height is generally insufficient to alter BMI category [29]. Respondents were additionaly asked to self-report their waist circumference as a secondary predictor of health risk [8], in an attempt to mitigate bias, particularly as it is known that BMI alone fails to differentiate muscle mass and fat mass [7]. It is also known that people poorly monitor their weight, particularly men, a contributing factor in self-reporting bias [28]. Web-based testing however, has been found to moderate socially desirable responses 1.51 times greater than comparable pen-and-pencil questionnaires when seeking a disclosure of sensitive issues, due largely to the anonymous nature of inquiry [30].

## Conclusion

People from cities, rural areas, health professionals and non-health professionals are equally challenged to accurately perceive a BMI corresponding with obesity in themselves and others. Weight status misperception was more likely to exist among those with a BMI less than 18.5 or 30 or more (underweight and obese). Largely, as weight status increased, accurate perception decreased. Visual cues however, appear to improve accuracy, given respondent corresponding with an obese BMI were significantly more likely to accurately self-perceive their weight status using silhouettes than medicalised words. Silhouettes therefore may act as an effective visual cue in initiating weight related discussions.

## Additional file

Additional file 1: Perceptions of body weight and preferred language. (PDF 53 kb)

## Data Availability

Both the original questionnaire and de-identified copies of completed questionnaires are available from the corresponding author. Data is available from the corresponding author on reasonable request.

## Abbreviations

BMI: Body Mass Index
CVD: Cardiovascular Disease
GP: General Practitioner
HP: Health Professional
MMM: Modified Monash Model
T2DM: Type 2 Diabetes Mellitus
