# Supplementary material for: Is social exposure to obesity associated with weight status misperception? Assessing Australians ability to identify overweight and obesity
Source: BMC Public Health. 2019 Sep 4;19:1222. doi: 10.1186/s12889-019-7556-9 (PMC6727423; doi:10.1186/s12889-019-7556-9)
Supplement: Supplementary file 1 — Perceptions of body weight and preferred language. (PDF 53 kb) [file 12889_2019_7556_MOESM1_ESM.pdf]

My First Instrument

Record ID ________________________________

What is your age? ________________________________

Do you identify as:
- ◯ Male
- ◯ Female
- ◯ Transgender/Intersex/Other

What is your height? ________________________________

What is your weight? ________________________________

What is your waist circumference? ________________________________

What is your postcode? ________________________________

In your opinion, do you think you are:
- ◯ Underweight
- ◯ Healthy Weight
- ◯ Overweight
- ◯ Obese

Are you a health professional?
- ◯ Yes
- ◯ No

How would you describe your employment status?
- ◯ Employed Full time
- ◯ Employed Part time
- ◯ Unemployed
- ◯ Student
- ◯ Retired
- ◯ Home duties

What is the highest level of education that you have completed?
- ◯ Partial secondary school
- ◯ Secondary school
- ◯ TAFE or trade school
- ◯ University

Has a doctor ever recommended that you lose weight?
- ◯ Yes
- ◯ No

In your opinion, should a doctor have recommended that you lose weight?
- ◯ Yes
- ◯ No
- ◯ In the past, but not now

If a doctor needed to speak with you about you needing to lose weight, how acceptable would you find each of these words or phrases?

Weight
- ◯ I would feel very upset or uncomfortable using this word/phrase to discuss my weight.
- ◯ I would feel mildly upset or uncomfortable using this word/phrase to discuss my weight.
- ◯ This word/phrase does not have an effect on me.
- ◯ I would feel comfortable using this word/phrase to discuss my weight.
- ◯ I would prefer to use this word/phrase to discuss my weight.

Body mass index (BMI)

- ◯ I would feel very upset or uncomfortable using this word/phrase to discuss my weight.
- ◯ I would feel mildly upset or uncomfortable using this word/phrase to discuss my weight.
- ◯ This word/phrase does not have an effect on me.
- ◯ I would feel comfortable using this word/phrase to discuss my weight.
- ◯ I would prefer to use this word/phrase to discuss my weight.

Unhealthy body weight

- ◯ I would feel very upset or uncomfortable using this word/phrase to discuss my weight.
- ◯ I would feel mildly upset or uncomfortable using this word/phrase to discuss my weight.
- ◯ This word/phrase does not have an effect on me.
- ◯ I would feel comfortable using this word/phrase to discuss my weight.
- ◯ I would prefer to use this word/phrase to discuss my weight.

Unhealthy body mass index (BMI)

- ◯ I would feel very upset or uncomfortable using this word/phrase to discuss my weight.
- ◯ I would feel mildly upset or uncomfortable using this word/phrase to discuss my weight.
- ◯ This word/phrase does not have an effect on me.
- ◯ I would feel comfortable using this word/phrase to discuss my weight.
- ◯ I would prefer to use this word/phrase to discuss my weight.

Obesity or Obese

- ◯ I would feel very upset or uncomfortable using this word/phrase to discuss my weight.
- ◯ I would feel mildly upset or uncomfortable using this word/phrase to discuss my weight.
- ◯ This word/phrase does not have an effect on me.
- ◯ I would feel comfortable using this word/phrase to discuss my weight.
- ◯ I would prefer to use this word/phrase to discuss my weight.

Weight problem

- ◯ I would feel very upset or uncomfortable using this word/phrase to discuss my weight.
- ◯ I would feel mildly upset or uncomfortable using this word/phrase to discuss my weight.
- ◯ This word/phrase does not have an effect on me.
- ◯ I would feel comfortable using this word/phrase to discuss my weight.
- ◯ I would prefer to use this word/phrase to discuss my weight.

Large size

- ◯ I would feel very upset or uncomfortable using this word/phrase to discuss my weight.
- ◯ I would feel mildly upset or uncomfortable using this word/phrase to discuss my weight.
- ◯ This word/phrase does not have an effect on me.
- ◯ I would feel comfortable using this word/phrase to discuss my weight.
- ◯ I would prefer to use this word/phrase to discuss my weight.

Excess fat

- ◯ I would feel very upset or uncomfortable using this word/phrase to discuss my weight.
- ◯ I would feel mildly upset or uncomfortable using this word/phrase to discuss my weight.
- ◯ This word/phrase does not have an effect on me.
- ◯ I would feel comfortable using this word/phrase to discuss my weight.
- ◯ I would prefer to use this word/phrase to discuss my weight.

Fatness or Fat

- ◯ I would feel very upset or uncomfortable using this word/phrase to discuss my weight.
- ◯ I would feel mildly upset or uncomfortable using this word/phrase to discuss my weight.
- ◯ This word/phrase does not have an effect on me.
- ◯ I would feel comfortable using this word/phrase to discuss my weight.
- ◯ I would prefer to use this word/phrase to discuss my weight.

Overweight Status or Overweight

- ◯ I would feel very upset or uncomfortable using this word/phrase to discuss my weight.
- ◯ I would feel mildly upset or uncomfortable using this word/phrase to discuss my weight.
- ◯ This word/phrase does not have an effect on me.
- ◯ I would feel comfortable using this word/phrase to discuss my weight.
- ◯ I would prefer to use this word/phrase to discuss my weight.

Your weight may be damaging your health

- ◯ I would feel very upset or uncomfortable using this word/phrase to discuss my weight.
- ◯ I would feel mildly upset or uncomfortable using this word/phrase to discuss my weight.
- ◯ This word/phrase does not have an effect on me.
- ◯ I would feel comfortable using this word/phrase to discuss my weight.
- ◯ I would prefer to use this word/phrase to discuss my weight.

You are overweight

- ◯ I would feel very upset or uncomfortable using this word/phrase to discuss my weight.
- ◯ I would feel mildly upset or uncomfortable using this word/phrase to discuss my weight.
- ◯ This word/phrase does not have an effect on me.
- ◯ I would feel comfortable using this word/phrase to discuss my weight.
- ◯ I would prefer to use this word/phrase to discuss my weight.

You need to lose some weight

- ◯ I would feel very upset or uncomfortable using this word/phrase to discuss my weight.
- ◯ I would feel mildly upset or uncomfortable using this word/phrase to discuss my weight.
- ◯ This word/phrase does not have an effect on me.
- ◯ I would feel comfortable using this word/phrase to discuss my weight.
- ◯ I would prefer to use this word/phrase to discuss my weight.

You are suffering from obesity	◯ I would feel very upset or uncomfortable using this word/phrase to discuss my weight.<br>◯ I would feel mildly upset or uncomfortable using this word/phrase to discuss my weight.<br>◯ This word/phrase does not have an effect on me.<br>◯ I would feel comfortable using this word/phrase to discuss my weight.<br>◯ I would prefer to use this word/phrase to discuss my weight.
You are obese	◯ I would feel very upset or uncomfortable using this word/phrase to discuss my weight.<br>◯ I would feel mildly upset or uncomfortable using this word/phrase to discuss my weight.<br>◯ This word/phrase does not have an effect on me.<br>◯ I would feel comfortable using this word/phrase to discuss my weight.<br>◯ I would prefer to use this word/phrase to discuss my weight.
You are heavier than you should be	◯ I would feel very upset or uncomfortable using this word/phrase to discuss my weight.<br>◯ I would feel mildly upset or uncomfortable using this word/phrase to discuss my weight.<br>◯ This word/phrase does not have an effect on me.<br>◯ I would feel comfortable using this word/phrase to discuss my weight.<br>◯ I would prefer to use this word/phrase to discuss my weight.
You are an unhealthy weight	◯ I would feel very upset or uncomfortable using this word/phrase to discuss my weight.<br>◯ I would feel mildly upset or uncomfortable using this word/phrase to discuss my weight.<br>◯ This word/phrase does not have an effect on me.<br>◯ I would feel comfortable using this word/phrase to discuss my weight.<br>◯ I would prefer to use this word/phrase to discuss my weight.
You are too fat	◯ I would feel very upset or uncomfortable using this word/phrase to discuss my weight.<br>◯ I would feel mildly upset or uncomfortable using this word/phrase to discuss my weight.<br>◯ This word/phrase does not have an effect on me.<br>◯ I would feel comfortable using this word/phrase to discuss my weight.<br>◯ I would prefer to use this word/phrase to discuss my weight.
You are above your healthy weight range	◯ I would feel very upset or uncomfortable using this word/phrase to discuss my weight.<br>◯ I would feel mildly upset or uncomfortable using this word/phrase to discuss my weight.<br>◯ This word/phrase does not have an effect on me.<br>◯ I would feel comfortable using this word/phrase to discuss my weight.<br>◯ I would prefer to use this word/phrase to discuss my weight.
Comments:	______________________________

Female body silhouettes

[Attachment: "Figure-1-Womens-BSG-images-weight-classifications-and-perceptions-by-respondents.png"]

Male body silhouettes

[Attachment: "Figure-2-Mens-BSG-images-weight-classifications-and-perceptions-by-respondents-Weight.png"]

In your opinion, which silhouette best matches your body?

◯ A
◯ B
◯ C
◯ D
◯ E
◯ F
◯ G
◯ H
◯ I
◯ J

In your opinion, which silhouettes are underweight?

☐ A
☐ B and below
☐ C and below
☐ D and below
☐ E and below
☐ F and below
☐ G and below
☐ H and below
☐ I and below
☐ J and below

In your opinion, which silhouettes are healthy weight?

☐ A
☐ B
☐ C
☐ D
☐ E
☐ F
☐ G
☐ H
☐ I
☐ J

In your opinion, which silhouettes are overweight?

◯ A
◯ B and above
◯ C and above
◯ D and above
◯ E and above
◯ F and above
◯ G and above
◯ H and above
◯ I and above
◯ J and above

In your opinion, which silhouettes are obese?

◯ A
◯ B and above
◯ C and above
◯ D and above
◯ E and above
◯ F and above
◯ G and above
◯ H and above
◯ I and above
◯ J and above
